# Rotavirus gastroenteritis hospitalizations in provinces with different vaccination coverage rates in Spain, 2013–2018

**DOI:** 10.1186/s12879-021-06841-x

**Published:** 2021-11-06

**Authors:** J. Ruiz-Contreras, S. Alfayate-Miguelez, B. Carazo-Gallego, E. Onís, L. Díaz-Munilla, M. Mendizabal, M. Méndez Hernández, B. Ferrer-Lorente, M. Unsaín-Mancisidor, J. T. Ramos-Amador, B. Croche-Santander, F. Centeno Malfaz, J. Rodríguez-Suárez, M. Cotarelo, M. San-Martín, J. Arístegui

**Affiliations:** 1 Pediatrics, Hospital Universitario 12 de Octubre, Madrid, Spain; 2 Pediatrics, Hospital Clínico Universitario Virgen de la Arrixaca, Murcia, Spain; 3 Pediatrics, Hospital Regional Universitario de Málaga, Malaga, Spain; 4 Pediatrics, Hospital Universitario de Basurto, Bilbao, Spain; 5 Pediatrics, Complejo Hospitalario de Navarra, Pamplona, Spain; 6 Pediatrics, Hospital Germans Trias i Pujol, Badalona, Spain; 7 Pediatrics, Hospital Universitario y Politécnico La Fe, Valencia, Spain; 8 Pediatrics, Hospital Universitario de Donostia, San Sebastián, Spain; 9 Pediatrics, Hospital Universitario Clínico San Carlos, Madrid, Spain; 10 Pediatrics, Hospital Juan Ramón Jiménez, Huelva, Spain; 11 Pediatrics, Hospital Universitario Río Hortega, Valladolid, Spain; 12 Pediatrics, Hospital Universitario Central de Asturias, Oviedo, Spain; 13 Medical Affairs Department, MSD Spain, C/Josefa Valcárcel, 38, 28027 Madrid, Spain

**Keywords:** Rotavirus, Acute Gastroenteritis, Hospitalization, Vaccination coverage rate, Spain

## Abstract

**Background:**

Rotavirus (RV) vaccines are available in Spain since 2006 but are not included in the National Immunization Program. RV vaccination has reached an intermediate vaccination coverage rate (VCR) but with substantial differences between provinces. The aim of this study was to assess the ratio of RV gastroenteritis (RVGE) admissions to all-cause hospitalizations in children under 5 years of age in areas with different VCR.

**Methods:**

Observational, multicenter, cross-sectional, medical record-based study. All children admitted to the study hospitals with a RVGE confirmed diagnosis during a 5-year period were selected. The annual ratio of RVGE to the total number of all-cause hospitalizations in children < 5 years of age were calculated. The proportion of RVGE hospitalizations were compared in areas with low (< 30%), intermediate (31–59%) and high (> 60%) VCR.

**Results:**

From June 2013 to May 2018, data from 1731 RVGE hospitalizations (16.47% of which were nosocomial) were collected from the 12 study hospitals. RVGE hospital admissions accounted for 2.82% (95 CI 2.72–3.00) and 43.84% (95% CI 40.53–47.21) of all-cause and Acute Gastroenteritis (AGE) hospitalizations in children under 5 years of age, respectively. The likelihood of hospitalization due to RVGE was 56% (IC95%, 51–61%) and 27% (IC95%, 18–35%) lower in areas with high and intermediate VCR, respectively, compared to the low VCR areas.

**Conclusions:**

RVGE hospitalization ratios are highly dependent on the RV VCR. Increasing VCR in areas with intermediate and low coverage rates would significantly reduce the severe burden of RVGE that requires hospital management in Spain.

*Clinical trial registration* Not applicable

**Supplementary Information:**

The online version contains supplementary material available at 10.1186/s12879-021-06841-x.

## Background

Rotavirus (RV) infection is a significant cause of hospitalization [1] in children, and is associated with an important consumption of health care resources [2, 3] representing a significant clinical and economic burden for the health care system in developed countries [4, 5]. RV has also been found to be a major etiologic agent for pediatric nosocomial AGE [6].

Before the availability of vaccines, RV was the leading cause of severe gastroenteritis in children < 5 year of age worldwide [1]. In Europe, it was estimated that every year RV accounted for 231 deaths, over 87,000 hospitalizations and almost 700,000 outpatients’ visits. A systematic review had confirmed the significant public health benefit of RV vaccination in Europe [2, 7]. Since 2009, World Health Organization Strategic Advisory Group of Experts (SAGE), recommended that all national immunization programs include RV vaccination for infants [8–10].

Two rotavirus vaccines have been available in Spain since 2006: RotaTeq^®^, (RV5; MSD) [11] and Rotarix^®^, (RV1; GSK) [12], but not included in the National Immunization Program. They are used under pediatricians’ recommendations and paid in full by parents, leading to an intermediate level of vaccination coverage and substantial differences between provinces [3, 13–19]. Both vaccines are indicated for immunization of infants from 6 weeks of age and the schedule must be completed before 24 or 32 weeks of age for Rotarix and RotaTeq, respectively. Because of the incidental finding of circovirus DNA contamination in both vaccines, the Spanish Medicine Agency suspended RV vaccine distribution temporarily, withdrawing RV5 from June 2010 to November 2010, and RV1 from February 2010 to June 2016 [13]. Since reintroduction after the 5 months suspension, VCR has steadily increased from 19% in 2010 to 52% in 2018.

Previous studies in Spain reported RVGE-related hospitalization rates that ranged between 120 and 480 cases per 100,000 children < 5 years of age [20–24]. This variability may be explained by some study-related factors, such as the selected populations, the observed study periods, and other methodological issues. Most of these studies are based on data collected from hospital administrative databases, which includes diagnostic information codified using the International Classification of Diseases (ICD) codes. The reliability of the estimation of burden of hospital-based disease depends, therefore, on the quality of codification. It has been stated that the use of hospital discharge databases frequently underestimate the incidence of RVGE-related hospitalizations, as a proportion of RV cases admitted to hospital is not coded as RVGE in the hospital discharge databases [13, 15–17, 21–25].

The objective of the present study was to estimate the annual ratio of RVGE hospitalizations among children under 5 years of age and to assess the differences in RVGE hospitalizations between areas with different VCR in Spain.

## Methods

### Study design

This was an observational, multicenter, cross-sectional study performed at the pediatric and microbiology departments of hospitals from provinces with different RV VCR in Spain. The VCR was estimated based on the number of RV vaccine doses distributed, provided by IQVIA (formerly IMS Health), and the number of newborns each year in the different provinces, obtained from the official census.

**Fig. 1:**
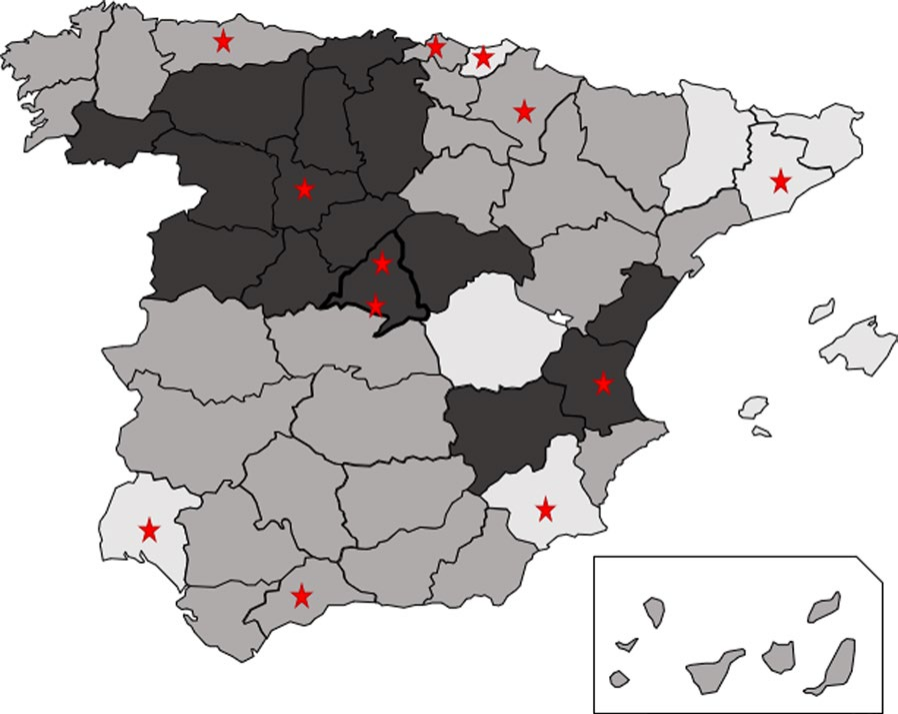
Distribution of study hospitals. Lighter color corresponds to low VCR provinces and darkest corresponds to high VCR provinces. Stars indicates the location of the study hospitals

According to the VCR observed in the different provinces of Spain (ranging from 10 to 75%, approximately), three study groups were defined: low (≤ 30%), intermediate (31–59%), and high (≥ 60%) VCR (Fig. 1).

Hospitals that performed systematic detection of RV in all children < 5 years admitted for AGE and with electronic records in the Pediatric and Microbiology Departments were considered for inclusion. The pediatric population (≤ 14 years of age) of the catchment area of each participating hospital was collected from hospital databases.

### Ethics

The study was designed, conducted and reported in accordance with the Guidelines for Good Pharmacoepidemiology Practices of the International Society for Pharmacoepidemiology [26], with the ethical principles of the Declaration of Helsinki, and with the current Spanish legislation related to observational studies (Ministerial Order SAS/3470/2009) [27]. The study was reviewed and approved by the Clinical Research Ethics Committee of the Basque Country in Txagorritxu Hospital (Vitoria, Spain).

### Study population

All children < 5 years of age hospitalized (admission to hospital for at least 24 h) from June 2013 to May 2018 with a microbiologically confirmed diagnosis of RVGE were selected for the analysis.

### Data collection

In each hospital, the microbiology department electronic records were examined to identify all hospitalizations with a positive laboratory test for RVGE in children < 5 years of age during the study period. The hospitals mainly used immunochromatography tests for RV detection, although the proportion of hospitals using immunochromatography versus molecular tests (PCR) increased from 1 out of 12 in 2013–2014 to 4 in 2017–2018.

The total number of all-cause emergency room (ER) visits or hospitalizations and the number of AGE of any etiology hospitalizations in children < 5 years of age were collected from the hospital administrative database. Clinical records of patients were reviewed to collect data on the type of RVGE (community-acquired or nosocomial).

All study data were reviewed and introduced in an electronic case report form by the investigators.

### Statistical analysis

For the estimation of the hospitalization ratios, sample size was determined based on the results published by Orrico-Sánchez et al. in which, with a VCR that reached up to 42.13%, the proportion of RVGE cases among all hospitalizations in children < 5 years of age during the postvaccine introduction period was 1.9%. The study was performed in 20 hospitals from the Autonomous Community of Valencia over a 7-year period [15]. That represents approximately 20 RVGE hospitalizations per hospital and year. Therefore, for the present study, a convenience sample of 12 hospitals would provide a total of 1200 hospitalizations due to RVGE during the 5-year study period. This sample would allow us to estimate a proportion of 1.9% RVGE hospitalizations among all hospitalizations with a precision of 0.79% and a confidence level of 95%.

Annual hospitalization ratios were calculated according to the formula: Annual number of diagnosed RVGE hospitalizations among children < 5 years of age/Total number of all-cause hospitalizations among children < 5 years of age during the study period × 100.

The proportion of all-cause ER visits that were hospitalized due to any cause, AGE and RVGE among the study groups was also calculated. The number of ER visits was used as a proxy to estimate which proportion of children < 5 years of age attending ER are hospitalized due to any cause, AGE and RVGE.

We calculated 95% confidence intervals (95% CIs) by assuming hospitalization rates followed a normal distribution and thus used standardized tables to estimate upper and lower bounds. Hospitalization ratios and 95% CIs for community-acquired RVGE and nosocomial RVGE separately were also calculated. For this purpose, records of a randomized sample of 1000 patients were reviewed to estimate the percentage of episodes, among the total sample, that were due to nosocomial and community-acquired infection. Nosocomial RVGE was defined as any episode of AGE appearing from at least 48 h after admission to 72 h after hospital discharge with laboratory test positive for RV infection.

The proportions of all-cause hospitalizations that were due to RVGE and AGE were compared among the three study groups, using a Chi-square or Fisher exact test, and adjusted odds ratios (ORs) with respective 95% CIs were calculated. Low VCR group was used as reference group for comparisons.

All statistical analyses were performed using SAS version 9.4. A level of statistical significance of 0,0.05 was applied to all statistical tests.

## Results

Twelve hospitals (4 per VCR group) were included in the study (Fig. 1). The average estimated VCR in the low, intermediate, and high VCR groups was 21%, 47% and 69%, respectively (Additional file 1: Table S1). Aggregated pediatric population (children ≤ 14 years of age) of the catchment area of hospitals was 278,197 children in the low, 271,192 children in the intermediate and 234,350 children in the high VCR groups.

A total of 1,347,770 all-cause ER visits, 61,448 all-cause hospitalizations, and 3972 AGE hospitalizations of any etiology in children < 5 years of age were collected during the 5-year study period. The total number of RVGE confirmed hospitalizations was 1731. The percentage of nosocomial RV infections among all RVGE-related hospitalizations was 16.47%.

Table 1 summarized the number and proportion of all-cause ER visits, all-cause hospitalizations, AGE and RVGE hospitalizations in children < 5 years of age per VCR group.

**Table 1:** Number and proportion of hospitalizations due to any cause, AGE and RVGE in children < 5 years of age over all-cause ER visits and hospitalizations during the study period (2013–2018) per VCR group

VCR group	Any cause			AGE			RVGE		
	Number of ER visits	Number of Hospit	% Hospit/ER visits	Number of Hospit	% Hospit/ER visits due to any cause	% Hospit/Hospit due to any cause	Number of Hospit	% Hospit/ER visits due to any cause	% Hospit/Hospit due to any cause
≤ 30%	507,750	19,401	3.82	1754	0.35	9.04	773	0.15	3.98
31–59%	395,394	17,856	4.52	1129	0.29	6.32	528	0.13	2.96
≥ 60%	444,626	24,191	5.44	1089	0.24	4.50	430	0.10	1.78
**Total**	**1,347,770**	**61,448**	**4.56**	**3972**	**0.29**	**6.46**	**1731**	**0.13**	**2.82**

*VCR* vaccination coverage rate, *AGE* Acute Gastroenteritis, *RVGE* Acute Gastroenteritis due to rotavirus, *Hospit* Hospitalization, *ER* emergency room

The high VCR group showed the highest proportion of hospitalizations due to any cause among children attending ER but the lowest proportion of hospitalizations due to RVGE (Table 1).

**Fig. 2:**
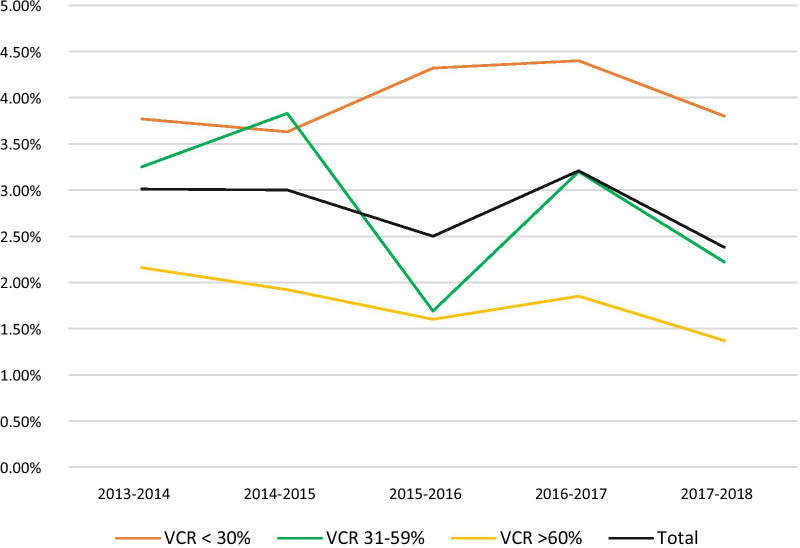
Annual proportion of all-cause hospitalization in children < 5 years that are due to RVGE among the VCR groups

The overall proportion of RVGE hospitalizations among all-cause hospitalizations in children < 5 years (including community-acquired and nosocomial) was 2.82% (95% CI 2.72–3.00) and ranged from 2.38% (95% CI 2.16–2. 26) to 3.21% (95% CI 2.95–3.49) in the different years included in the study period (Fig. 2). Different annual patterns can be observed in the different study groups. In the intermediate VCR group, biennial peaks are observed in the 2014–2015 and 2016–2017 seasons. A less pronounced peak is also observed during the 2016–2017 season in the high VCR group. RVGE accounted for 43.84% (95% CI 40.53–47.21) of the total AGE hospitalizations in children < 5 years of age, ranging from 40.39% (95% CI 37.33–43.51) to 49.87% (95% CI 46.83–52.91) in the study period years.

Among the study groups, the percentages of nosocomial infections were 14.07%, 20.66% and 14.67% for the low, intermediate, and high VCR groups, respectively. Community-acquired and nosocomial RVGE episodes accounted for 2.38% (95% CI 2.15–2,63) and 0.52% (95% CI 0.40–0.68), respectively, of all-cause hospitalizations in children < 5 years of age during the study period and 36.85% (95% CI 33.55–40.29) and 7.73% (95% CI 5.95–9.96), respectively, of AGE- hospitalizations.

Table 2 summarizes the estimated differences in the proportions of RVGE hospitalizations between study groups, considering the low VCR group as reference for comparisons.

**Table 2:** Proportions of all-cause hospitalizations in children < 5 years that are due to RVGE and OR (95% CI) during the study period among VCR groups

VCR group	Proportion of hospitalizations due to RVGE % (95% CI)	OR (95% CI)
≤ 30%	3.98 (3.71–4.27)	Ref
31–59%	2.96 (2.71–3.22)	0.73 (0.65–0.82)
≥ 60%	1.78 (1.62–1.95)	0.44 (0.39–0.49)

Proportions of hospitalizations were calculated among all-cause hospitalizations in children < 5 years

*VCR* vaccination coverage rate, *RVGE* Acute Gastroenteritis due to Rotavirus, *AGE* Acute Gastroenteritis, *OR* odds ratio, *95% CI* 95% confidence interval, *Ref.* reference group for OR calculation

Statistically significant differences were observed in the proportion of hospitalization due to RVGE in children < 5 years between study groups. Compared to the area with the lowest VCR (≤ 30%), the proportion of RVGE hospitalizations was 56% and 27% lower in the high and the intermediate VCR groups, respectively (Table 2).

## Discussion

Vaccines against RV have been shown to have an impact in reducing the hospitalizations due to RVGE in children < 5 years of age that seems to be highly dependent on the fulfilled vaccination coverage [13, 28].

We found an association between reductions in RVGE-related hospitalizations and RV VCR across all provinces studied. The greater the VCR, the larger the decline in the likelihood of RVGE hospitalizations, as showed by the 27% and 56% lower proportions of hospitalizations due to RVGE in the intermediate a high VCR group, respectively, compared to the low VCR area. Previous studies in the Valencia region have described this specific coverage-related impact on hospitalizations due to RVGE. Orrico-Sánchez et al. found an age-dependent RVGE hospitalization rate decrease of 67% to 71% with a VCR ≥ 40% compared to no vaccination. These reductions seem to be higher than those observed in our study, but it is important to note that the reference group for comparisons in our study was a low vaccination group rather than a no vaccination group; this could explain why the reductions in hospitalization ratios in our study were less pronounced. In the analysis conducted by Orrico-Sánchez, reduction in the RVGE hospitalization rate was observed in very young children even in areas with VCR of less than 20% [15]. These findings support the need to increase the vaccination coverage to maximize its clinical impact, especially on the severe burden of disease requiring hospital treatment.

The proportion of RVGE recorded as nosocomial in our study (16.5%), is consistent with the incidences reported by other authors [6, 29–31]. This may be considered of epidemiologic and clinical concern, and efforts to implement preventive measures, including vaccination, should be made.

Interestingly, hospitals in the high VCR group showed a greater proportion of pediatric all-cause hospital admissions over the total ER visits but the lowest RVGE hospitalization ratios. Considering only the time period analyzed in the study, this may suggests that the differences in the RVGE hospitalization ratios are related to the increase in the VCR for RV vaccines, regardless of the pediatric hospital admission capacity or the hospital-related criteria for admission for a given disease. More analyses, including ER visits and hospitalization data outside the vaccination period, should be performed to confirm this observation.

Another interesting finding in our study is that, the reductions in the RVGE hospitalizations seem to be stronger than the differences in the VCR between groups; For example, while the reduction in the proportion of RV hospitalization between high and low VCR groups was 57%, the difference between the average VCR between the same groups was 48%. Other authors have observed an indirect protective effect of the RV vaccination on unvaccinated individuals [32]. Anyhow, our study was not design to evaluate the indirect effects so attributing these results to herd protection should be made with caution.

During the study period, small biennial hospitalization ratios increases are observed in the intermediate and high VCR groups. This pattern has been previously reported in other countries, like the US, where vaccination coverage is suboptimal [33]. On the other hand, in Finland, in which vaccination coverage rates were over 90% since vaccine program implementation, low level of RV activity is observed with no biennial epidemics reported [34].

Our study has some limitations. First, other factors besides vaccination such as changes in the detection methods of RVGE episodes over time and differences in the admission criteria between hospitals may have contributed to the differences in hospitalization ratios observed [13]. More strict admission criteria may have contributed to the lower number of hospitalizations due to RVGE in the high VCR group, but in that case, other cause hospitalizations would have been probably lower also. Therefore, by using the total number of children < 5 years hospitalized due to any cause as denominator to calculate the hospitalization ratios minimizes the potential effect of differences in hospital admission criteria. Moreover, data aggregation from different hospitals in each study group would have also minimized the impact of individual hospital factors in the differences observed. Secondly, only hospitals with microbiological electronic records for the 5-year study period from provinces with different VCR were selected. Hence, the study population might not be entirely representative of the Spanish pediatric population. Finally, as RV vaccination is not included in the national immunizations program in Spain, no official vaccination coverage figures are available; therefore, for the purposes of this study, the number of vaccine doses distributed and the total number of children born each year within each province was used to estimate the VCR. Observed results depend on the VCR in the years before the study. Substantial changes in the prior VCR may have an impact on hospitalization rates estimated. In Spain, since 2010 VCR has been increasing in all provinces, but changes were steady with no significant fluctuations from 1 year to another.

In contrast, strengths include the long study period, including five RV seasons, the number and diversity of participating hospitals, the large sample size included in the analysis, and the use of reliable sources of data that allow the identification of all hospitalizations due RVGE.

## Conclusion

Ratios of RVGE-related hospitalizations are highly dependent on the RV VCR. Burden of hospitalizations due to RVGE is still important in Spain and may be reduced by increasing the vaccination coverage rate in areas with moderate vaccine use.

## Supplementary Information

**Additional file 1: Table S1.** List of the study hospitals and VCR of the province.

## Data Availability

The datasets used and/or analyzed during the current study are available from the corresponding author on reasonable request.

## Abbreviations

AGE: Acute Gastroenteritis
ER: Emergency room
OR: Odds ratios
RV: Rotavirus
RVGE: Rotavirus acute gastroenteritis
VCR: Vaccination coverage rates
