# Supplementary material for: Rotavirus gastroenteritis hospitalizations in provinces with different vaccination coverage rates in Spain, 2013–2018
Source: BMC Infect Dis. 2021 Nov 6;21:1138. doi: 10.1186/s12879-021-06841-x (PMC8572461; doi:10.1186/s12879-021-06841-x)
Supplement: Supplementary file 1 — Additional file 1: Table S1. List of the study hospitals and VCR of the province. [file 12879_2021_6841_MOESM1_ESM.docx]

**Table S1.** Hospitals per VCR group involved in the study

| VCR | Study Hospital (Province) | Estimated VCR during study period |
| --- | --- | --- |
| ≤ 30% | Hospital Donostia (Guipúzcoa)  Hospital General Juan Ramón Jiménez (Huelva)  Hospital Virgen de la Arrixaca (Murcia)  Hospital Germans Trias i Pujol (Barcelona) | 9%  16%  29%  30% |
| 31-59% | Hospital Basurto (Vizcaya)  Hospital Universitario Central de Asturias (Asturias)  Hospital Virgen del Camino (Navarra)  Complejo Hospitalario Regional de Málaga (Málaga) | 39%  46%  47%  57% |
| ≥60% | Hospital Clínico San Carlos (Madrid)  Hospital Universitario 12 de Octubre (Madrid)  Hospital La Fe (Valencia)  Hospital Río Hortega (Valladolid) | 67%  67%  72%  73% |

VCR: Vaccination coverage rate

Based on vaccine doses distribution provided by IQVIA (formerly (IMS Health)
